# Mobile robot path planning with reformative bat algorithm

**DOI:** 10.1371/journal.pone.0276577

**Published:** 2022-11-04

**Authors:** Gongfeng Xin, Lei Shi, Guanxu Long, Weigang Pan, Yiming Li, Jicun Xu

**Affiliations:** 1 Innovation Research Institute, Shandong Hi-speed Group Co. LTD, Jinan, Shandong, China; 2 School of Information Science and Electrical Engineering (School of Artificial Intelligence), Shandong Jiaotong University, Jinan, Shandong, China; National Taiwan University of Science and Technology, TAIWAN

## Abstract

Mobile robot path planning has attracted much attention as a key technology in robotics research. In this paper, a reformative bat algorithm (RBA) for mobile robot path planning is proposed, which is employed as the control mechanism of robots. The Doppler effect is applied to frequency update to ameliorate RBA. When the robot is in motion, the Doppler effect can be adaptively compensated to prevent the robot from prematurely converging. In the velocity update and position update, chaotic map and dynamic disturbance coefficient are introduced respectively to enrich the population diversity and weaken the limitation of local optimum. Furthermore, Q-learning is incorporated into RBA to reasonably choose the loudness attenuation coefficient and the pulse emission enhancement coefficient to reconcile the trade-off between exploration and exploitation, while improving the local search capability of RBA. The simulation experiments are carried out in two different environments, where the success rate of RBA is 93.33% and 90%, respectively. Moreover, in terms of the results of success rate, path length and number of iterations, RBA has better robustness and can plan the optimal path in a relatively short time compared with other algorithms in this field, thus illustrating its validity and reliability. Eventually, by the aid of the Robot Operating System (ROS), the experimental results of real-world robot navigation indicate that RBA has satisfactory real-time performance and path planning effect, which can be considered as a crucial choice for dealing with path planning problems.

## Introduction

As the representative of high-end intelligent equipment and high-tech, mobile robot technology is changing with each passing day, which has been widely applied in family services, rescue and relief, warehousing and logistics, and other practical application fields. In order to achieve the shortest collision-free movement of the mobile robot from the starting point to the target point, the path planning of the mobile robot has become a hot spot of current research, and has attracted close attention of relevant scholars. To date, a variety of effective methods have been developed to deal with path planning problems, such as visibility graph [1, 2], artificial potential field (APF) [3, 4], rapidly-exploring random tree (RRT) [5], reinforcement learning (RL) [6], ReinforcedRimJump (RRJ) [7, 8], nonlinear control [9], etc. Nevertheless, with the increase of environment complexity and task difficulty, the above path planning methods are hard to achieve desired effects. The path drawn by the visibility graph or the RRT is composed of multiple straight lines, resulting in the path is not smooth enough. The APF is easy to get trapped into local optima, moreover the phenomenon that the target point is unreachable may occur. For the RL, it is difficult to use less resources to address the path planning problem in complex environments. As an emerging algorithm, RRJ can achieve the shortest path planning, but it is only suitable for static environment, which seriously affects its practical application value.

Since the establishment of swarm intelligence (SI) [10], it has become a research field of great concern, bringing hope to solve complex optimization problems. The inspiration of SI mainly comes from the collective behavior patterns of ants, bees, bats, and other biological groups. All of these creatures search their targets through common wisdom and experience. The SI-based optimization algorithms simulate the behavioral attributes of biological populations, including particle swarm optimization (PSO) [11, 12], teaching-learning-based optimization (TLBO) [13, 14], artificial bee colony (ABC) optimization [15–17], ant colony optimization (ACO) [18–23], firefly algorithm (FA) [24, 25], bat algorithm (BA) [26–31], whale optimization algorithm (WOA) [32], etc. As a classic swarm intelligence optimization algorithm, PSO is frequently utilized to handle mobile robot path planning problems due to its simple structure, high search efficiency, and easy improvement. So far, many valuable research results have emerged [33–36]. Mo and Xu [33] proposed a novel approach for global path planning in a static environment that hybridizes biogeography-based optimization (BBO) and PSO. Tang et al. [34] introduced a hybrid PSO that combines PSO and differential evolution (DE) algorithm. Mac et al. [35] conducted a more in-depth study on the path planning problem of mobile robots in complex environments and presented a constrained multi-objective PSO. However, the above PSO variants are prone to fall into local optima, making it difficult to efficiently complete optimal path planning. Li and Chou [36] came up with a SLPSO algorithm and comprehensively considered constraints such as path length, collision risk degree and smoothness to generate a feasible collision-free path. Nevertheless, the robustness of the algorithm is not satisfactory, that is, as the complexity of the environment increases, the path planning effect of the algorithm declines.

The BA, first introduced in 2010, is similar to PSO, however, it has better convergence and can balance exploration and exploitation well when searching for the global optimum. Consequently, BA is increasingly favored by researchers, and a number of well-known BA variants have been advanced one after another. Liu et al. [37] put forward a modified BA, called PTRBA, to process the global path planning problem of single-robot or multi-robots. In order to improve the optimization performance of BA, the dynamic perturbation coefficient is introduced into the position update in the global search stage, and the tangent random exploration mechanism is integrated into the local search stage. Eventually, the PTRBA and cubic spline interpolation are combined to form a smooth and feasible path. In reference [38], an adaptive robotic bat algorithm (ARBA) was put forth to handle the multirobot target searching problem. The adaptive inertial weight strategy is added to the velocity update to improve the diversity of ARBA. Furthermore, the Doppler effect and multi-swarm strategy are introduced into ARBA to assist robots to better accomplish target searching. Based on the above description, BA variants have many merits, but there are still some challenges to be solved. For instance, loudness attenuation coefficient and pulse emission enhancement coefficient are the key elements that influence the balance between exploration and exploitation of BA. If the above two parameters are not properly coordinated, the optimization performance of BA will be affected, making it hard to guarantee the path planning effect. However, the BA variants described above do not take this factor into account. Hence, there is still plenty of room for improvement in their performance.

In order to further improve BA and better complete the path planning task in static environments, this paper puts forward a reformative BA, named RBA, in which all robots are regarded as bats, and one robot represents one bat. Moreover, RBA is employed as the robots^’^ control mechanism to realize the robots’ search for the target, thereby accomplishing the path planning task. The main contributions of RBA are highlighted in the following aspects: (1) The Doppler effect is applied to the frequency update to ameliorate RBA. When the robot is in motion, the Doppler effect can be adaptively compensated to prevent the robot from prematurely converging. (2) In the velocity update and position update, chaotic map and dynamic disturbance coefficient are introduced respectively to enrich the population diversity and weaken the limitation of local optimum. (3) Q-learning is adopted to make reasonable choices for the loudness attenuation coefficient and the pulse emission enhancement coefficient to coordinate the trade-off between exploration and exploitation, while improving the local search capability of RBA. To verify the validity and reliability of RBA, simulation experiments are carried out in two different environments. To begin with, the original RBA is compared with five classical swarm intelligence optimization algorithms, including PSO, BA, FA, TLBO and WOA. The experimental results demonstrate that RBA has good comprehensive performance and can effectively and reliably implement the optimal path planning. Subsequently, RBA is compared with four PSO variants, namely BPSO [33], PSO-DE [34], CMOPSO [35] and SLPSO [36]. Experimental results show that contrasted with PSO variants, RBA has superior search performance and stronger robustness. Finally, the proposed RBA is compared with three other state-of-the-art BA variants, i.e. EBat [28], PTRBA [37] and ARBA [38]. Experimental results indicate that RBA can give consideration to optimization effect and computational efficiency, and has excellent robustness. With the help of ROS, real-world robot navigation experiments are also carried out. The related results reveal that RBA has satisfactory real-time performance and path planning effect, and can be considered as a crucial choice for dealing with path planning problems.

The remainder of this paper is organized as follows. In ‘Bat algorithm’ and ‘Q-learning’, we review the knowledge of BA and Q-learning, respectively. The proposed RBA is described in detail in ‘Reformative bat algorithm (RBA)’. To evaluate the proposed approach, simulation experiments are conducted in ‘Simulation testing’ and real-world robot navigation experiments are finished in ‘Real-world case’. In the end, conclusions are drawn and future work is provided in ‘Conclusions and future work’.

## Bat algorithm

BA was first introduced in 2010, inspired by bats’ echolocation behavior in search of prey. In nature, bats emit ultrasonic pulses and analyze reflected ultrasonic waves to determine the information of prey. Besides, bats can search for prey by changing their ultrasonic frequency, velocity and position. In the process of approaching prey, bats will increase the emissivity of ultrasonic pulses and weaken the loudness. The implementation of BA is based on the following assumptions. (1) All bats use echolocation to sense distance, and they can accurately distinguish between prey and obstacles. (2) Bats can automatically adjust the frequency and emissivity of the pulses according to the proximity of the target. (3) It is assumed that the loudness changes from a maximum value to a fixed minimum value.

The frequency, velocity and position values of each bat can be calculated as

$$\begin{array}{c} f_{i}=f_{min}+\left(f_{max}-f_{min}\right)\cdot \beta , \end{array}$$ (1)

$$\begin{array}{c} v_{i}^{t}=v_{i}^{t-1}+\left(x_{i}^{t}-x^{*}\right)\cdot f_{i}, \end{array}$$ (2)

$$\begin{array}{c} x_{i}^{t}=x_{i}^{t-1}+v_{i}^{t}, \end{array}$$ (3)

where *f*_*max*_ and *f*_*min*_ are the maximum and minimum values of the search pulse frequency, respectively; *β* ∈ [0, 1] is a uniformly distributed random number; x* indicates the optimal position of all current bats.

For the local search stage, a new result is performed in accordance with the following:

$$\begin{array}{c} x_{new}=x_{old}+\epsilon A^{t}, \end{array}$$ (4)

where *x*_*old*_ is the current best solution, *x*_*new*_ is the new solution generated after the local search; *ϵ* ∈ [−1, 1] is a random number; *A*^*t*^ is the average loudness of all bats at iteration *t*.

The iterative equations for loudness *A*_*i*_ and pulse emissivity *r*_*i*_ are expressed as follows:

$$\begin{array}{c} A_{i}^{t+1}=\alpha A_{i}^{t}, \end{array}$$ (5)

$$\begin{array}{c} r_{i}^{t+1}=r_{i}^{0}\cdot [1-\text{exp}\;(-\gamma t)], \end{array}$$ (6)

where *α* and *γ* are constants; $r_{i}^{0}$ is the initial pulse emissivity. For any 0 < *α* < 1 and *γ* > 0, we have $A_{i}^{t}\to 0$, $r_{i}^{t}\to r_{i}^{0}$, as *t* → + ∞.

The pseudo code of BA is listed in Algorithm 1. As can be seen from Algorithm 1, the pulse emissivity *r*_*i*_ controls whether BA can perform local search, and the loudness *A*_*i*_ determines the local search performance of BA. Furthermore, according to Eqs (5) and (6), it is distinct that the loudness attenuation coefficient *α* and the pulse emission enhancement coefficient *γ* play a vital role in the iterative process of loudness and pulse emissivity, respectively. Therefore, in order to effectively coordinate the balance between exploration and exploitation and improve the local search capability of BA, it is necessary to reasonably choose the loudness attenuation coefficient and the pulse emission enhancement coefficient. In this paper, Q-learning is employed to tackle this issue. The details will be given in ‘Parameters preselection’.

## Q-learning

Q-learning is a trial and error learning method, whose purpose is to learn optimal strategies to accumulate rewards, so as to maximize the Q-value. The Q-value is updated as follows:

$$\begin{array}{c} Q\left(s_{t},a_{t}\right)\leftarrow \left(1-\mu \right)Q\left(s_{t},a_{t}\right)+\mu [re\left(s_{t},a_{t}\right)+\eta \underset{a_{t+1}}{\text{max}}\;Q\left(s_{t+1},a_{t+1}\right)], \end{array}$$ (7)

where *re*(*s*_*t*_, *a*_*t*_) is an immediate reward; *η* is a discount factor; *μ* is the learning rate, which controls the learning speed. Within a certain range of values, the larger the *μ*, the faster the convergence.

In this paper, greedy strategy is chosen as action selection strategy. The greedy strategy, as the name implies, aims to select the action that maximizes the Q-value. The relevant equation is expressed as

$$\begin{array}{c} a_{t}=\text{arg}\;\underset{a_{t}}{\text{max}}\;Q\left(s_{t},a_{t}\right). \end{array}$$ (8)

## Reformative bat algorithm (RBA)

As mentioned in ‘Introduction’, BA has both advantages and challenges. Thus, in this section, the RBA is proposed to address the corresponding challenges and significantly improve the BA. On the one hand, the Doppler effect, chaotic map and dynamic disturbance coefficient are utilized to assist RBA to avoid premature convergence and weaken the limitation of local optimum. On the other hand, by means of Q-learning, RBA can effectively solve the challenges of BA caused by the poor coordination between loudness attenuation coefficient and pulse emission enhancement coefficient.

**Algorithm 1** Pseudo code of BA.

Determine the fitness function *fit*(*x*), *x* = (*x*_1_, *x*_2_, ⋯, *x*_*d*_)^*T*^;

Generate bat population *x*_*i*_ (*i* = 1, 2, ⋯, *m*) and initial velocity *v*_*i*_ (*i* = 1, 2, ⋯, *m*);

Define pulse frequency *f*_*i*_ at *x*_*i*_;

Initialize values for pulse emissivity *r*_*i*_ and loudness *A*_*i*_;

**while** *t* ≤ *T*_*max*_ **do**

 Adjust frequency by Eq (1);

 Update velocities by Eq (2);

 Update positions by Eq (3);

 **if** *rand* > *r*_*i*_ **then**

  Select a best position;

  Generate a local position by Eq (4);

 **end**

 **if** *rand* < *A*_*i*_ *and* *fit*(*x*_*i*_) > *fit*(x*) **then**

  Accept the new position;

  Update *A*_*i*_ by Eq (5);

  Update *r*_*i*_ by Eq (6);

 **end**

 Find the current best x*;

 **if** *target is reached or stop condition is met* **then**

  break;

 **end**

**end**

Show the results

### Doppler effect

According to Eq (1), we can intuitively see that the frequency update of BA has a strong randomness, resulting in the planned path is not smooth enough, and premature convergence may occur. Consequently, the Doppler effect is introduced to ameliorate the frequency update of BA. The improved frequency calculation formula is expressed as

$$\begin{array}{c} f_{i}=f_{min}+(f_{max}-f_{min})\cdot \xi_{i}, \end{array}$$ (9)

$$\begin{array}{c} \xi_{i}=(\frac{v\pm v_{i}^{t}}{v\mp v_{s}})\cdot \xi_{0}, \end{array}$$ (10)

where *ξ*_*i*_ is the observation frequency, *ξ*_0_ is the original emission frequency of the emission source (target); *v* is the velocity of wave propagation; $v_{i}^{t}$ is the movement velocity of the observer (robot), if the observer is close to the emission source, the operator in front is “+”, otherwise it is “-”; *v*_*s*_ is the movement velocity of the emission source, if the emission source is close to the observer, the operator in front is “-”, otherwise it is “+”.

In the light of Eq (10), we can discover that in the Doppler effect, the frequency will change as the distance between the robot and the target changes. Hence, the robot can adaptively compensate for the Doppler effect during the movement, and then regulate the velocity by adaptively adjusting the frequency, thereby avoiding premature convergence.

### Improved model for velocity and position

In RBA, the velocity and position values can be updated as

$$\begin{array}{c} v_{i}^{t}=v_{i}^{t-1}+\sigma \cdot \left(x_{i}^{t}-x^{*}\right)\cdot f_{i}, \end{array}$$ (11)

$$\begin{array}{c} \sigma =\sigma_{t}=\zeta \cdot \text{sin}\;\left(\pi \cdot \sigma_{t-1}\right),\;\sigma_{t}\in \left(0,1\right),\;t=1,2,\cdots ,T_{max}, \end{array}$$ (12)

$$\begin{array}{c} x_{i}^{t}=\omega \cdot x_{i}^{t-1}+v_{i}^{t}, \end{array}$$ (13)

$$\begin{array}{c} \omega =1-\text{sin}\;(\frac{\pi t}{2\cdot T_{max}})+\tau \cdot betarnd\left(\right). \end{array}$$ (14)

The standard BA uses Eq (3) to update the position, in which the calculation of $v_{i}^{t}$ is inseparable from $x_{i}^{t}-x^{*}$. Hence, when conducting the global search, BA is directly constrained by $x_{i}^{t}-x^{*}$, and it is easy to fall into local optima. In response to this problem, the attenuation coefficient *σ* is introduced in Eq (12). Since chaotic map has the merits of ergodicity, non-repeatability and sensitivity, we select chaotic map to update *σ*, where *ζ* ∈ (0, 1) is a constant and *t* represents the current iteration number. Based on Eq (12), it is evident that the value range of *σ* always belongs to (0, 1). Therefore, the limitation of local optimum is reduced. In addition, the dynamic disturbance coefficient *ω* is put forward as shown in Eq (14), where *τ* is the disturbance deviation factor and *betarnd*() is a random number obeying the beta distribution. The dynamic disturbance coefficient *ω* decreases adaptively with the increase of the number of iterations. Consequently, in the early stage, the dynamic disturbance coefficient *ω* has a large disturbance to the position update, which is conducive to expanding the search scope of bats. In the later stage, the dynamic disturbance coefficient *ω* reduces the disturbance to the position update, which is beneficial to the stability of the algorithm. Through many experiments, the constant *ζ* and the disturbance deviation factor *τ* are set to 0.5 and 0.1, respectively.

### Parameters preselection

**Fig 1:**
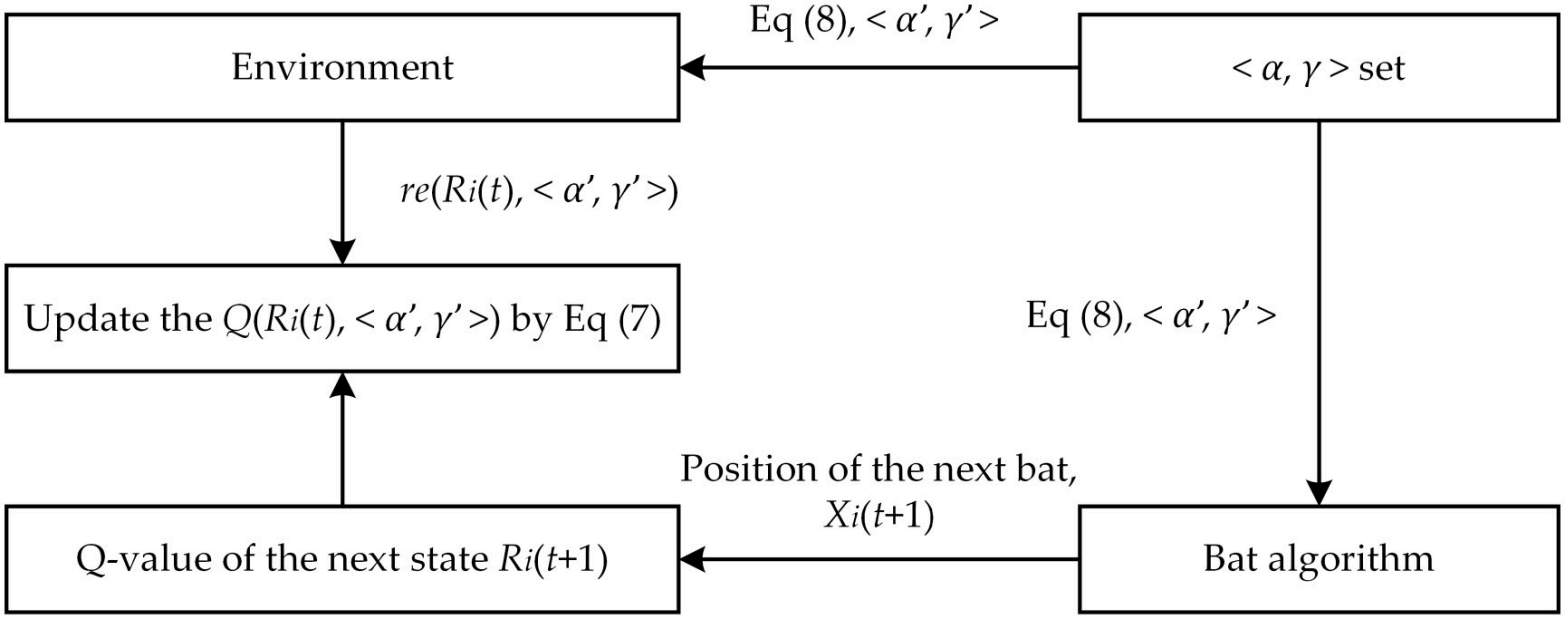
The combination of bat algorithm and Q-learning.

In BA, the quality of optimization results is determined by loudness attenuation coefficient *α* and pulse emission enhancement coefficient *γ*. If the above parameters are not properly coordinated, the convergence speed of BA will be affected, making it difficult to ensure the path planning effect. Therefore, in the local search phase, Q-learning is applied to preselect the optimal combinations of the above parameters to ameliorate the optimization effect of BA. The relevant idea is displayed in Fig 1.

In Fig 1, < *α*, *γ* > set is composed of the loudness attenuation coefficient *α* and the pulse emission enhancement coefficient *γ*, and a < *α*, *γ* > combination corresponds to an action in Q-learning. *X*_*i*_(*t*) is defined as the position of the *i*th bat at iteration *t*. Moreover, *R*_*i*_(*t*) is the fitness function value of the bat at position *X*_*i*_(*t*), which is defined as the state of Q-learning. The combination of BA and Q-learning can be described as selecting the optimal combination < *α*′, *γ*′ > from the < *α*, *γ* > set according to Eq (8) when the state is *R*_*i*_(*t*). In BA, the optimal combination < *α*′, *γ*′ > is utilized to obtain the next position *X*_*i*_(*t* + 1) of the bat, and then the Q-value of the next state *R*_*i*_(*t*+ 1) is estimated. On the other hand, when the optimal action < *α*′, *γ*′ > acts on the environment, the corresponding immediate reward *re*(*R*_*i*_(*t*), < *α*′, *γ*′ >) will be generated. The immediate reward is set to the difference between the fitness function values of the bats in successive iterations. The related equation is executed as follows:

$$\begin{array}{c} re(R_{i}\left(t\right),<\alpha^{\prime },\gamma^{\prime }>)=fit(X_{i}\left(t+1\right))-fit(X_{i}\left(t\right))=R_{i}\left(t+1\right)-R_{i}\left(t\right). \end{array}$$ (15)

Finally, *Q*(*R*_*i*_(*t*), <*α*′, *γ*′>) is updated in accordance with Eq (7).

Owing to the application of the Q-learning, in the local search phase, each bat position has its corresponding optimal < *α*′, *γ*′ > combination, and all the information is saved in the Q-table. In the implementation stage, RBA can directly select the optimal < *α*′, *γ*′ > combinations from the Q-table, thus overcoming the defects of the standard BA due to the parameters are not well coordinated.

### Fitness function

In this paper, the fitness function is designed in the light of the following evaluation criteria. (1) No collision with obstacles. (2) Achieve the shortest path length. The corresponding fitness function is expressed as

$$\begin{array}{c} fit=\frac{1}{L\cdot (1+\bar{p}\cdot \lambda )}, \end{array}$$ (16)

where *L* is the path length of the mobile robot from the starting point to the target point, which conforms to Eq (17),

$$\begin{array}{c} L=\sum_{i=1}^{n}\sqrt{{\left(x_{i+1}-x_{i}\right)}^{2}+{\left(y_{i+1}-y_{i}\right)}^{2}}. \end{array}$$ (17)

$\bar{p}$ is the penalty term used to exclude paths that collide with obstacles. The value of $\bar{p}$ is set to 100. λ is the flag variable with an initial value of 0. The update process of λ is as follows:

*for k* = 1: *nobs*

$$\begin{array}{ccc} & & \;d_{k}=\sqrt{{\left(xx-xobs_{k}\right)}^{2}+{\left(yy-yobs_{k}\right)}^{2}}, \end{array}$$ (18)

$$\begin{array}{ccc} & & \;\theta_{k}=\text{max}\;(1-\frac{d_{k}}{robs_{k}},0), \end{array}$$ (19)

$$\begin{array}{ccc} & & \;\lambda =\lambda +mean(\theta_{k}). \end{array}$$ (20)

*end*

Given that the robot has a certain volume, the obstacles are expanded to prevent the robot from hitting the obstacles. *nobs* is the total number of obstacles. (*xobs*_*k*_, *yobs*_*k*_) and *robs*_*k*_ are the center coordinate and maximum influence radius of the *k*th expanded obstacle, respectively. *d*_*k*_ is the distance from the point on the path to the center coordinate of the obstacle. For λ, if there is no collision between the robot and the obstacle, then λ = 0. However, if the robot collides with the obstacle, λ is a positive number greater than 0. Hence, when the fitness function *fit* reaches the maximum value, the shortest collision-free path can be obtained.

### Implementation of RBA

After model improvement and parameters preselection, RBA will be implemented into path planning. In the global search stage, the Doppler effect, attenuation coefficient and dynamic disturbance coefficient are added to the RBA. Consequently, unlike standard BA, the frequency, velocity and position values in RBA are updated according to Eqs (9)–(14). In the local search stage, RBA can directly select the corresponding optimal <*α*′, *γ*′> combination from the Q-table on the basis of the current position of the bat, which can significantly improve the optimization performance of the algorithm.

The pseudo code of RBA is given in Algorithm 2.

**Algorithm 2**: Pseudo code of RBA.

Determine the fitness function *fit*(*x*), *x* = (*x*_1_, *x*_2_, ⋯, *x*_*d*_)^*T*^;

Generate bat population *x*_*i*_ (*i* = 1, 2, ⋯, *m*) and initial velocity *v*_*i*_ (*i* = 1, 2, ⋯, *m*);

Define pulse frequency *f*_*i*_ at *x*_*i*_;

Initialize values for pulse emissivity *r*_*i*_ and loudness *A*_*i*_;

**while** *t* ≤ *T*_*max*_ **do**

 Adjust frequency by Eqs (9) and (10);

 Update velocities by Eqs (11) and (12);

 Update positions by Eqs (13) and (14);

 **if** *rand* > *r*_*i*_ **then**

  Select a best position;

  Select the optimal combination < *α*′, *γ*′ > from the Q-table;

  Generate a local position by Eq (4);

 **end**

 **if** *rand* < *A*_*i*_ and *fit*(*x*_*i*_) > *fit*(x*) **then**

  Accept the new position;

  Update *A*_*i*_ by Eq (5);

  Update *r*_*i*_ by Eq (6);

 **end**

 Find the current best x*;

 **if** *target is reached or stop condition is met* **then**

  break;

 **end**

**end**

Show the results

### Complexity analysis of RBA

The time computational complexity of the proposed RBA can be expressed as *O*((*T* + *M*_1_) × (*N* + *M*_2_) × *D*), where *T* is the number of iterations, *N* is the population size, *D* is the dimension of the path planning problem to be addressed, *M*_1_ is the computation time of the attenuation coefficient *σ* and dynamic disturbance coefficient *ω*, and *M*_2_ indicates the computation time for the observation frequency *ξ*_*i*_. For the original BA, its time computational complexity can be described as *O*(*T* × *N* × *D*). From Eqs (10), (12) and (14), it is apparent that only simple numerical operations are involved in *M*_1_ and *M*_2_. Hence, the time computational complexity of the proposed RBA is only slightly increased compared to that of BA.

## Simulation testing

### Experimental setup

In order to verify the validity and feasibility of the proposed RBA, five classical swarm intelligence optimization algorithms (PSO, BA, FA, TLBO and WOA), four PSO variants (BPSO, PSO-DE, CMOPSO and SLPSO), and three BA variants (EBat, PTRBA and ARBA) are selected to compare with RBA. To ensure the objectivity and fairness of the algorithm comparison, all experiments are conducted in Windows 10 environment, using Intel(R) Core(TM) i7–8750H 2.2GHz CPU and 8GB RAM, and all algorithms are implemented in MATLAB R2018b. Two static maps of different complexity are constructed, in which the number of obstacles is 9 and 13, respectively, as shown in Figs 5(a) and 9(a). The scale of both maps is 10 × 10, where the yellow square and green star are the starting point and the target point, respectively. We run the proposed RBA and baseline algorithms 30 times on each map and calculate the mean and standard deviation of the experimental results for comparison. The experimental results include the path length planned by each algorithm and the iteration number required by each algorithm to complete the path planning.

Based on previous researches, the key parameters of the above algorithms are fairly chosen as follows. The population size and the maximum number of iterations are set to *npop* = 100 and *T*_*max*_ = 100, respectively. For PSO, BPSO, PSO-DE, CMOPSO and SLPSO, inertia weight and acceleration coefficients are selected as *ω* = 1 and *c*_1_ = *c*_2_ = 1.5, respectively. Especially, in PSO-DE, scaling factor *F* = 0.5 and crossover rate *CR* = 0.5. For FA, randomization parameter *α* = 0.5, light absorption coefficient *γ* = 1, highest attractiveness *β*_0_ = 2 and constant *m* = 2. In BA, EBat, PTRBA and ARBA, the numerical settings of the loudness attenuation coefficient and the pulse emission enhancement coefficient are the same, i.e. *α* = *γ* = 0.9. Different from BA, EBat, PTRBA and ARBA, RBA selects the optimal *α* and *γ* values from the Q-table.

### Test case 1

The map used in test case 1 contains nine obstacles. The shortest collision-free path on this map is shown in Fig 5(a), where (0, 0) is the starting point, (8, 10) is the target point, and the optimal path length is approximately 13.1716.

#### Comparison with classical algorithms

**Fig 2:**
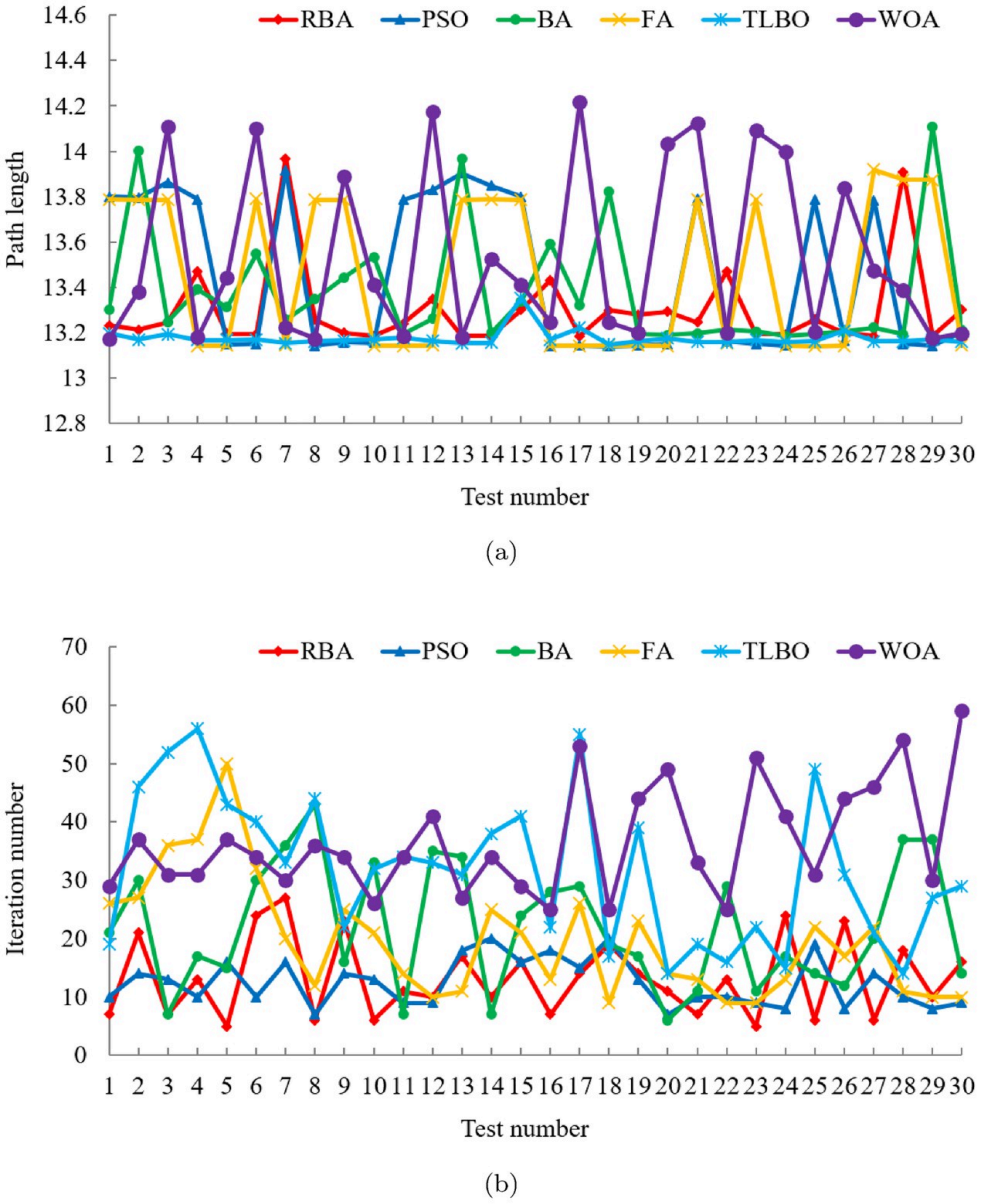
Experimental results of six algorithms in test case 1. (a) Path length. (b) Iteration number.

Five classical optimization algorithms are compared with our approach to demonstrate the superiority of the proposed RBA. In order to objectively analyze the performance of the algorithms and avoid contingency, each algorithm runs 30 times on the map. After 180 experiments, the experimental results are shown in Fig 2. In the 30 experiments of each algorithm, RBA realizes the optimal path 28 times, PSO realizes the optimal path 17 times, BA realizes the optimal path 26 times, FA realizes the optimal path 16 times, TLBO realizes the optimal path 30 times, and WOA realizes the optimal path 20 times. Consequently, the success rates of the above algorithms are 93.33%, 56.67%, 86.67%, 53.33%, 100%, and 66.67%, respectively.

For an in-depth understanding of the distribution of the experimental data in Fig 2, the mean and standard deviation of the relevant data are listed in Table 1. According to Fig 2(a) and Table 1, the path length data curve of TLBO is very stable. This is because in 30 experiments, TLBO has planned the optimal path every time, which also confirms that TLBO has the best ability to search for the global optimum. Excluding TLBO algorithm, among the remaining algorithms, it is clear that RBA has better performance compared with other algorithms, and its path length data curve shows relatively small fluctuations. In the light of Table 1, it is obvious that the average path length of RBA is 13.3019, and the standard deviation of the path length is only 0.1913. In terms of the number of iterations, as can be seen intuitively from Fig 2(b) and Table 1, the average number of iterations required for PSO to accomplish the path planning is the least, which is 12.43. The second is RBA, with an average number of iterations of 13.2. Although PSO can fulfill the path planning quickly, it has the defect that it is easy to fall into the local optima and cannot plan the optimal path effectively. This can be verified from the optimal path planning success rate, Fig 2(a) and Table 1. Relative to the excellent performance in path length, TLBO does not perform satisfactorily in the number of iterations. In 30 experiments, the average number of iterations of TLBO is 31.8, and the standard deviation of the number of iterations is as high as 12.7344.

**Table 1:** Performance comparison between RBA and classical optimization algorithms in test case 1.

Algorithm	RBA	PSO	BA	FA	TLBO	WOA
Average Path Length	13.3019	13.4432	13.3805	13.4543	13.1762	13.5402
Standard Deviation of Path Length	0.1913	0.3391	0.2654	0.3386	0.0373	0.3905
Average Iteration Number	13.2	12.43	21.87	19.6	31.8	36.67
Standard Deviation of Iteration Number	6.6974	4.0402	10.7663	9.9848	12.7344	9.4989

Furthermore, under the same experiment, the path planning results of the six algorithms are displayed in Fig 5(b). It is obvious that PSO, BA and FA are all trapped in local optima, and only RBA, TLBO and WOA plan the shortest path. Among them, in order to complete the optimal path planning, TLBO requires 8 iterations, WOA requires 60 iterations, while RBA only requires 5 iterations. Therefore, contrasted with the classical optimization algorithms, RBA can achieve the optimal path planning in a relatively short time, and the success rate can reach 93.33%, which demonstrates that RBA has the merits of rapid optimization speed and good optimization effect.

#### Comparison with PSO variants

**Fig 3:**
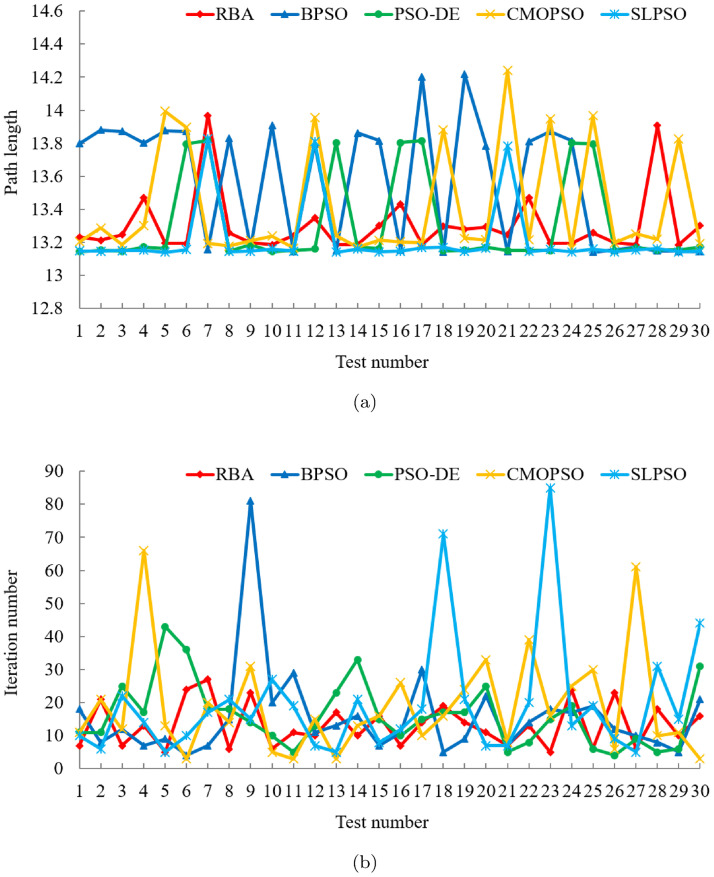
Experimental results of five algorithms in test case 1. (a) Path length. (b) Iteration number.

In order to compare the path planning effects of RBA and PSO variants, 150 experiments are fulfilled, and the related experimental results are exhibited in Fig 3. In the 30 experiments of each algorithm, RBA realizes the optimal path 28 times, BPSO realizes the optimal path 13 times, PSO-DE realizes the optimal path 23 times, CMOPSO realizes the optimal path 22 times, and SLPSO realizes the optimal path 27 times. Consequently, the success rates of the above algorithms are 93.33%, 43.33%, 76.67%, 73.33%, and 90%, respectively. On the basis of the success rate of each algorithm, it is obvious that in addition to SLPSO, other PSO variants have relatively poor performance and are prone to fall into local optimum, making it difficult to achieve optimal path planning.

To clearly analyze the experimental data in Fig 3, the mean and standard deviation of the corresponding data are shown in Table 2. It can be seen intuitively from Table 2 that the average path length of the five algorithms is roughly the same, while RBA has the smallest standard deviation of path length, which indicates that RBA has a more stable path planning effect. In terms of the number of iterations, RBA achieves the smallest mean and standard deviation values, which means that RBA can accomplish optimal path planning faster than PSO variants. Moreover, under the same experiment, the path planning results of the five algorithms are exhibited in Fig 5(c). Obviously, except BPSO, other algorithms plan the shortest path, among which, PSO-DE requires 9 iterations, CMOPSO requires 49 iterations, SLPSO requires 16 iterations, while RBA only requires 4 iterations.

**Table 2:** Performance comparison between RBA and PSO variants in test case 1.

Algorithm	RBA	BPSO	PSO-DE	CMOPSO	SLPSO
Average Path Length	13.3019	13.5662	13.3084	13.413	13.2171
Standard Deviation of Path Length	0.1913	0.3808	0.2787	0.3445	0.2003
Average Iteration Number	13.2	15.5	16.1333	18.8	19.4667
Standard Deviation of Iteration Number	6.6974	14.0755	9.8811	15.4661	18.2204

#### Comparison with BA variants

**Fig 4:**
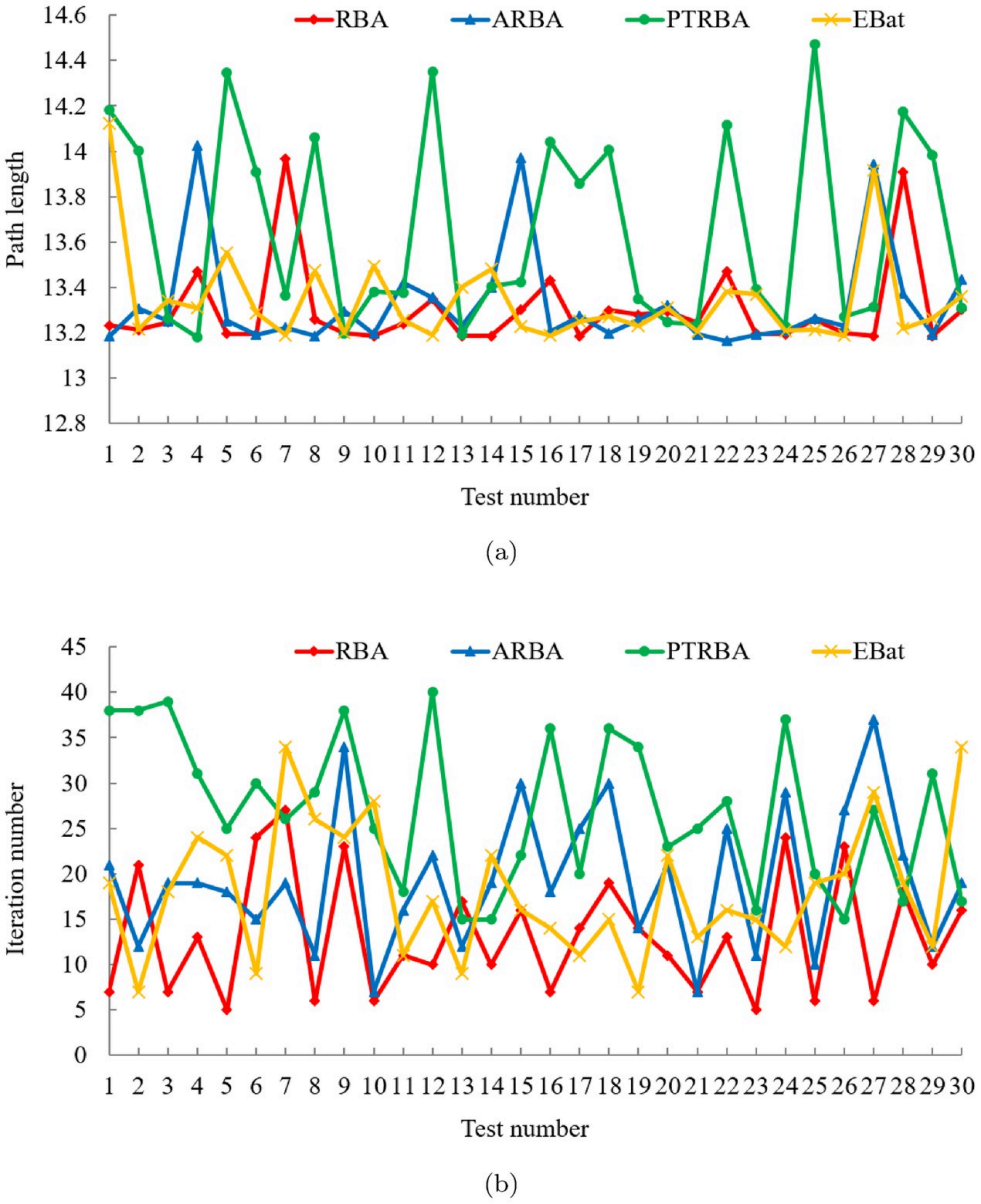
Experimental results of four algorithms in test case 1. (a) Path length. (b) Iteration number.

To finish the performance comparison between RBA and other novel BA variants, we collate 120 experimental data in Fig 4 and present the mean and standard deviation of the relevant data in Table 3. In the 30 experiments of each algorithm, RBA realizes the optimal path 28 times, ARBA realizes the optimal path 27 times, PTRBA realizes the optimal path 17 times, and EBat realizes the optimal path 28 times. Consequently, the success rates of the above algorithms are 93.33%, 90%, 56.67%, and 93.33%, respectively. Based on Fig 4 and Table 3, it is distinct that the path planning effect of PTRBA is relatively poor. In our opinion, this is because the tangent random exploration mechanism is applied in the local search phase of PTRBA, which replaces *ϵ* in Eq (4). The tangent random exploration mechanism is represented as tan(*π* ⋅ (*ξ* − 0.5)), where *ξ* is a random number belonging to [0, 1]. When the value of *ξ* approaches 0 or 1, the value of the tangent function approaches infinity. Therefore, in the iterative process of PTRBA, the phenomenon that the value of the tangent function is too large may occur, which influences the stability of the algorithm and reduces the path planning effect. For ARBA and EBat, their optimization performance is roughly the same and better than that of PTRBA. Compared with the aforementioned BA variants, RBA has more excellent path planning effects, not only in the path length but also in the number of iterations, thus verifying the superiority of RBA.

**Table 3:** Performance comparison between RBA and BA variants in test case 1.

Algorithm	RBA	ARBA	PTRBA	EBat
Average Path Length	13.3019	13.3313	13.6544	13.3442
Standard Deviation of Path Length	0.1913	0.2325	0.4308	0.2124
Average Iteration Number	13.2	19.37	27.03	18.13
Standard Deviation of Iteration Number	6.6974	7.757	8.3438	7.3472

**Fig 5:**
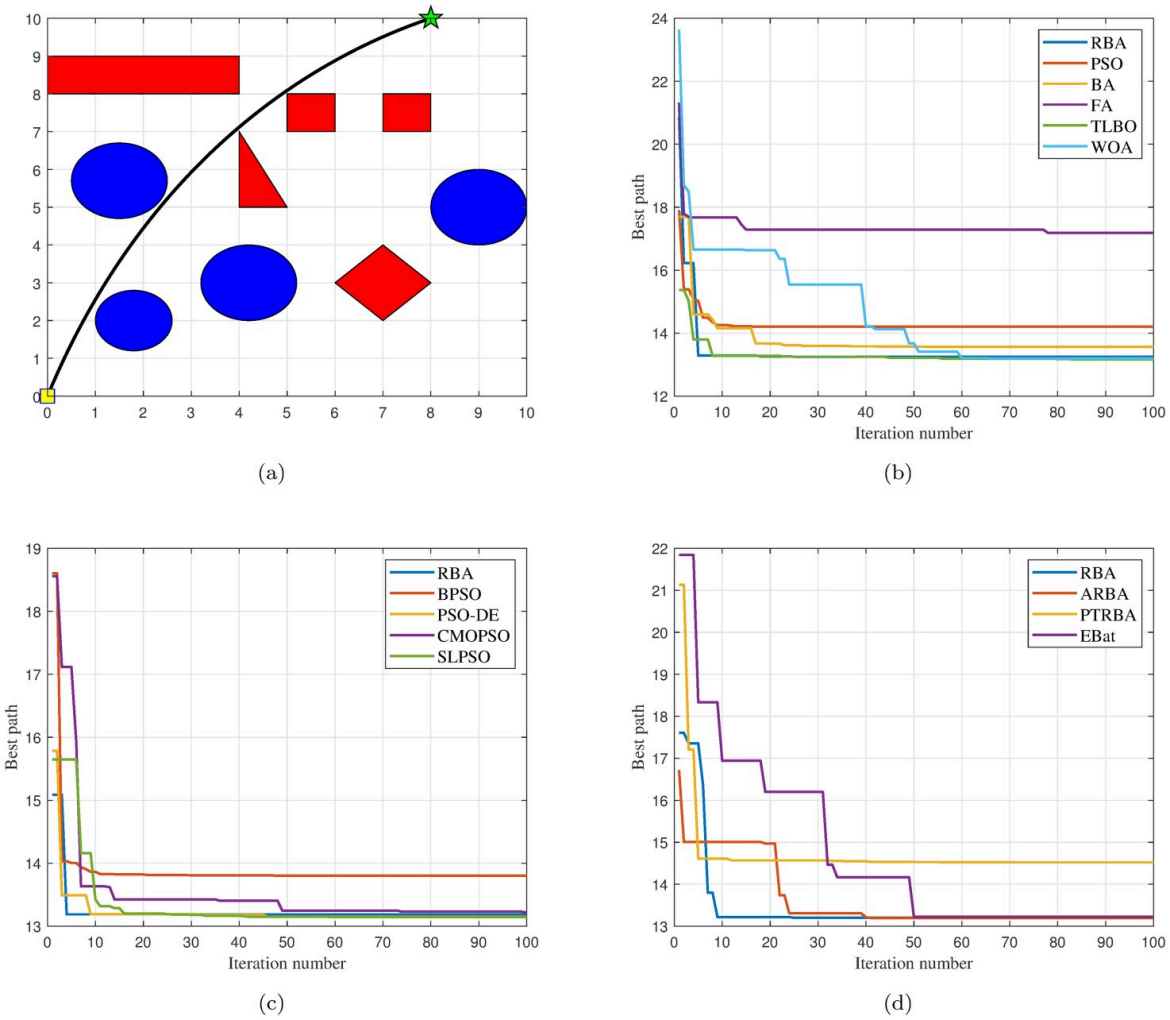
The path planning results in the test case 1 and the number of iterations of all algorithms when the path is implemented. (a) Optimal path. (b) Iteration curves of RBA and classical algorithms. (c) Iteration curves of RBA and PSO variants. (d) Iteration curves of RBA and BA variants.

Besides, under the same experiment, the path planning results of the four algorithms are displayed in Fig 5(d). As the above analysis of the shortcomings of PTRBA, although PTRBA converges quickly, it plans a relatively long path and has poor optimization effect. In contrast with PTRBA, other algorithms plan the shortest path, among which, ARBA requires 40 iterations, EBat requires 50 iterations, while RBA only requires 9 iterations.

### Test case 2

In order to further demonstrate the superiority of RBA, a more complex map is used in test case 2, which contains thirteen obstacles. The shortest collision-free path on this map is drawn in Fig 9(a), where (0, 0) is the starting point, (8, 10) is the target point, and the optimal path length is approximately 13.1966.

#### Comparison with classical algorithms

**Fig 6:**
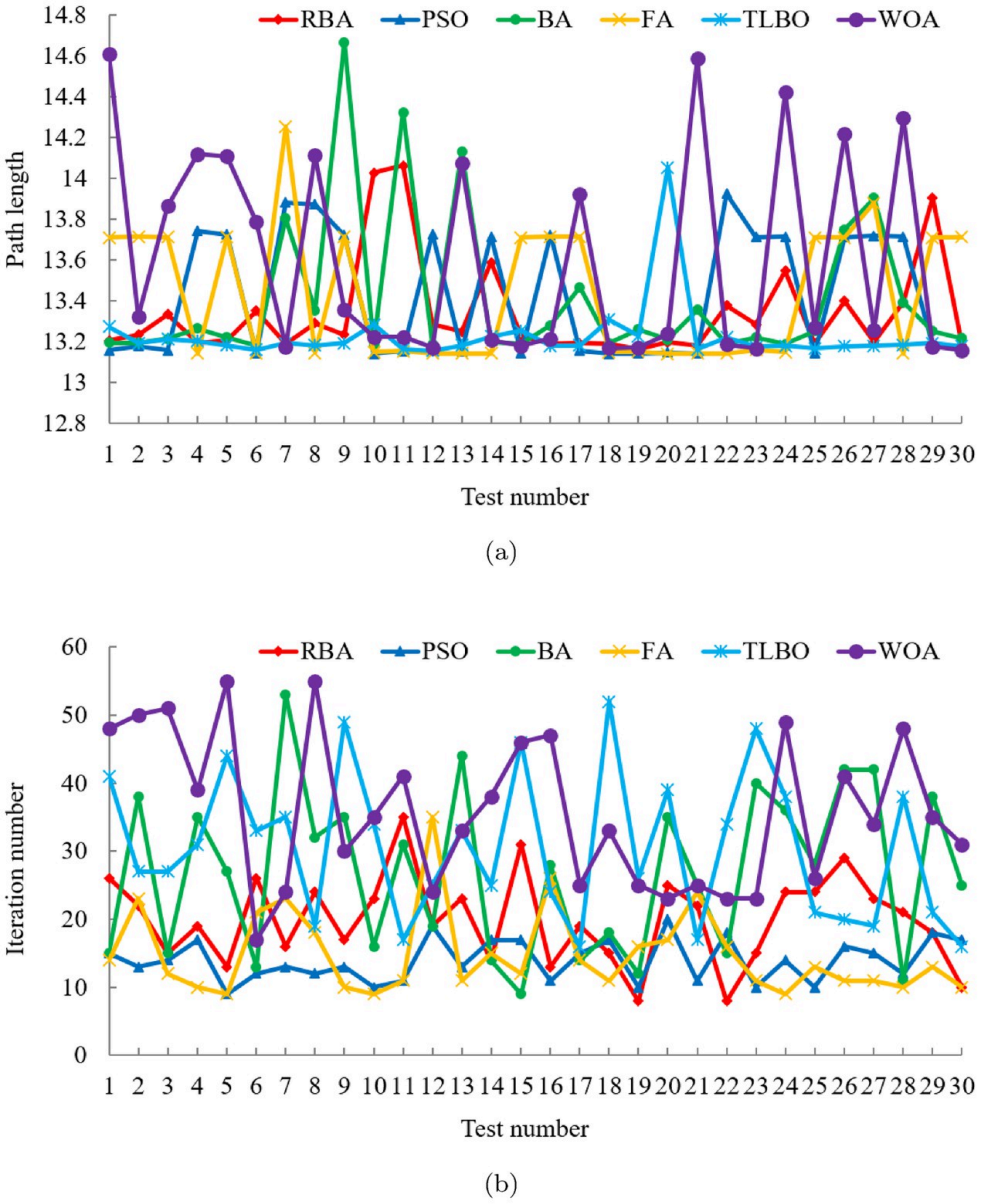
Experimental results of six algorithms in test case 2. (a) Path length. (b) Iteration number.

In the comparison experiment between RBA and five classical optimization algorithms, after 180 experiments, the experimental results are shown in Fig 6. In addition, the mean and standard deviation of the related experimental results are listed in Table 4. In the 30 experiments of each algorithm, RBA realizes the optimal path 27 times, PSO realizes the optimal path 16 times, BA realizes the optimal path 24 times, FA realizes the optimal path 16 times, TLBO realizes the optimal path 29 times, and WOA realizes the optimal path 18 times. Consequently, the success rates of the above algorithms are 90%, 53.33%, 80%, 53.33%, 96.67%, and 60%, respectively. For RBA, compared to the results of test case 1, the performance is slightly decreased. The probability of realizing the optimal path is reduced by 3.33%.

**Table 4:** Performance comparison between RBA and classical optimization algorithms in test case 2.

Algorithm	RBA	PSO	BA	FA	TLBO	WOA
Average Path Length	13.3436	13.436	13.4159	13.4352	13.2287	13.6005
Standard Deviation of Path Length	0.2469	0.3111	0.3819	0.3281	0.1603	0.5077
Average Iteration Number	19.9	13.97	26.83	14.83	30.5	35.8
Standard Deviation of Iteration Number	6.5303	3.0904	12.0146	6.1928	10.7855	11.1213

On the basis of Table 4, it is clear that the average path length of RBA is 13.3436, and the standard deviation of the path length is 0.2469. Excluding TLBO algorithm, the RBA performs better than other algorithms. In terms of the number of iterations, PSO and FA can accomplish the path planning task faster than RBA. However, they are difficult to achieve the optimal path planning, and the success rate of optimal path planning is relatively low. Moreover, under the same experiment, the path planning results of the six algorithms are depicted in Fig 9(b). It is distinct that only RBA, BA, and TLBO fulfill the shortest path planning. Among them, in order to implement the optimal path, BA requires 10 iterations, TLBO requires 27 iterations, while RBA only requires 8 iterations. Thus, contrasted with classical optimization algorithms, RBA has good overall performance, not only achieves good path planning effect, but also has satisfactory robustness.

#### Comparison with PSO variants

**Fig 7:**
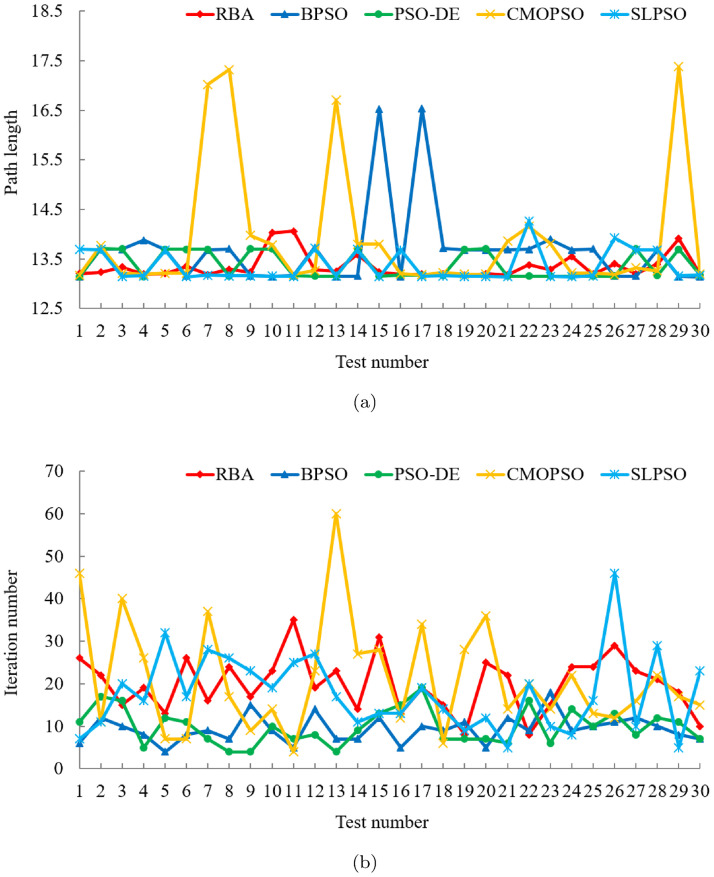
Experimental results of five algorithms in test case 2. (a) Path length. (b) Iteration number.

To compare the optimization performance of RBA and PSO variants, 120 experiments are conducted and the experimental data are presented in Fig 7. In the 30 experiments of each algorithm, RBA realizes the optimal path 27 times, BPSO realizes the optimal path 12 times, PSO-DE realizes the optimal path 18 times, CMOPSO realizes the optimal path 18 times, and SLPSO realizes the optimal path 20 times. Consequently, the success rates of the above algorithms are 90%, 40%, 60%, 60%, and 66.67%, respectively. For PSO-DE, CMOPSO and SLPSO, the performance is significantly degraded compared to the results in test case 1. The success rates of the three algorithms are reduced by 16.67%, 13.33%, and 23.33%, respectively.

The mean and standard deviation of the experimental results in Fig 7 are exhibited in Table 5. It can be seen from Fig 7 and Table 5 that in terms of the number of iterations, in addition to CMOPSO, BPSO, PSO-DE and SLPSO can complete the path planning faster than RBA. Nevertheless, in the light of the success rate and path length results, PSO variants, especially BPSO and CMOPSO, have unsatisfactory global optimization performance and are prone to fall into local optima. Besides, under the same experiment, the path planning results of the five algorithms are depicted in Fig 9(c). It is clear that only RBA and SLPSO accomplish the shortest path, among which, SLPSO requires 14 iterations, while RBA only requires 7 iterations.

**Table 5:** Performance comparison between RBA and PSO variants in test case 2.

Algorithm	RBA	BPSO	PSO-DE	CMOPSO	SLPSO
Average Path Length	13.3436	13.6786	13.3749	13.9078	13.3586
Standard Deviation of Path Length	0.2469	0.8235	0.2692	1.3125	0.313
Average Iteration Number	19.9	9.3	9.87	21.23	17.7
Standard Deviation of Iteration Number	6.5303	3.153	4.1501	13.0481	9.1544

#### Comparison with BA variants

**Fig 8:**
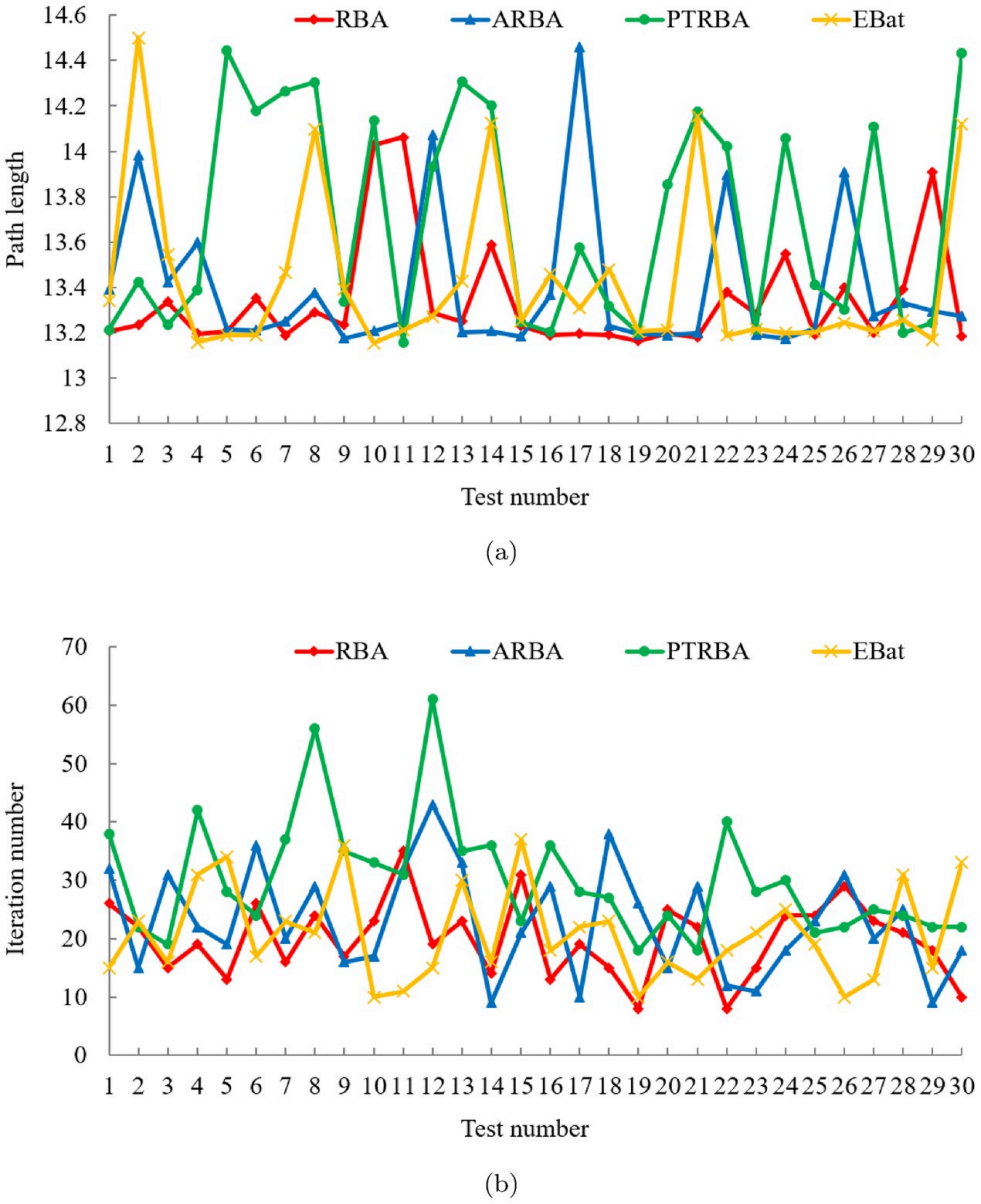
Experimental results of four algorithms in test case 2. (a) Path length. (b) Iteration number.

After 120 experiments, we collate the experimental results of RBA and other state-of-the-art BA variants in Fig 8. Meanwhile, the mean and standard deviation of the relevant experimental data are listed in Table 6. In the 30 experiments of each algorithm, RBA realizes the optimal path 27 times, ARBA realizes the optimal path 25 times, PTRBA realizes the optimal path 16 times, and EBat realizes the optimal path 25 times. Consequently, the success rates of the above algorithms are 90%, 83.33%, 53.33%, and 83.33%, respectively.

**Table 6:** Performance comparison between RBA and BA variants in test case 2.

Algorithm	RBA	ARBA	PTRBA	EBat
Average Path Length	13.3436	13.3984	13.703	13.4314
Standard Deviation of Path Length	0.2469	0.3278	0.4682	0.3699
Average Iteration Number	19.9	22.97	30.17	20.73
Standard Deviation of Iteration Number	6.5303	9.1594	10.2959	8.1238

**Fig 9:**
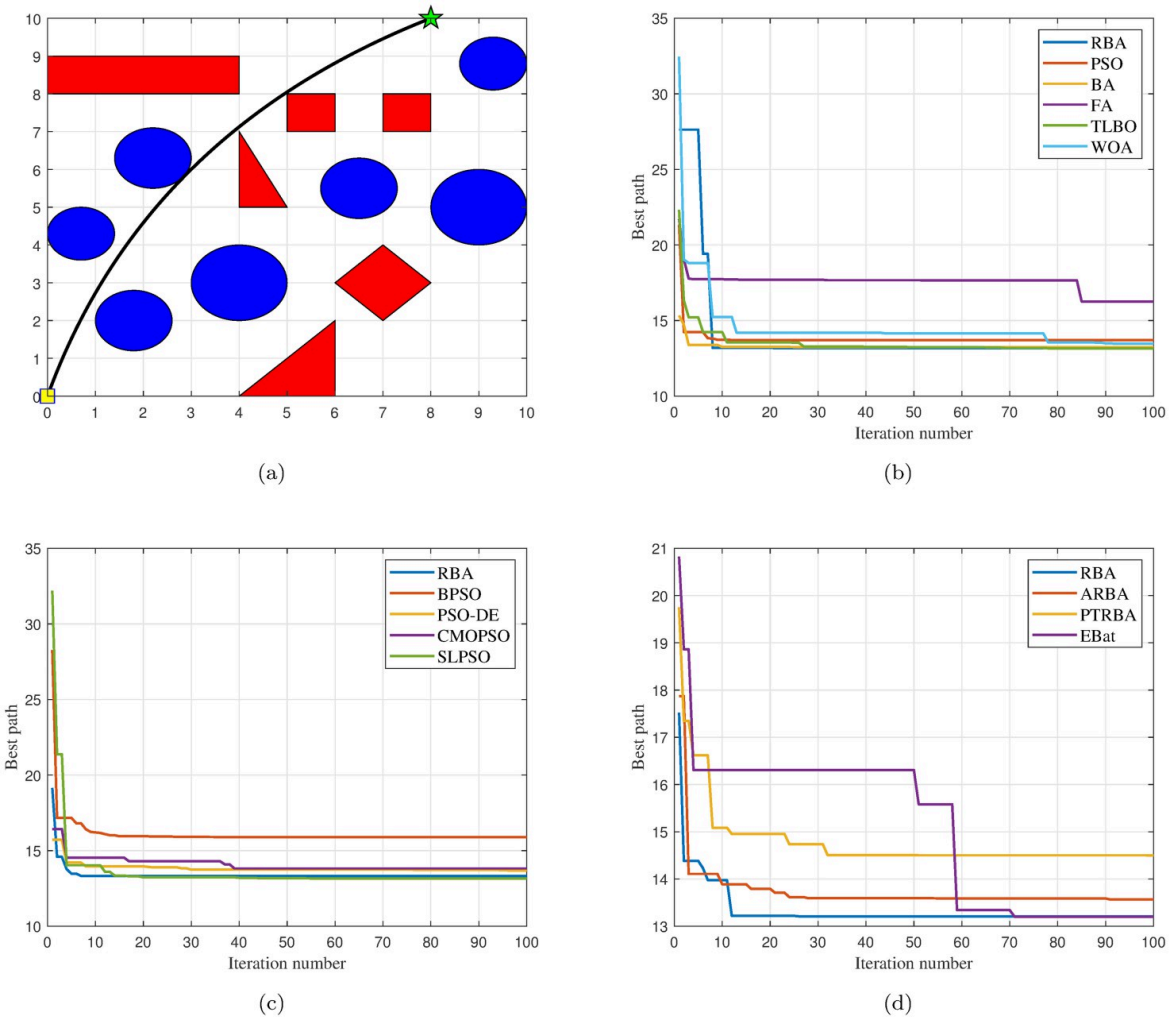
The path planning results in the test case 2 and the number of iterations of all algorithms when the path is implemented. (a) Optimal path. (b) Iteration curves of RBA and classical algorithms. (c) Iteration curves of RBA and PSO variants. (d) Iteration curves of RBA and BA variants.

On the basis of Table 6, we can get that compared with the novel BA variants, RBA has better path planning effect, not only in the path length but also in the number of iterations, which further proves the superiority of RBA. Furthermore, under the same experiment, the path planning results of the four algorithms are displayed in Fig 9(d). It is evident that only RBA and EBat fulfill the shortest path planning, among which, EBat requires 71 iterations, while RBA only requires 12 iterations.

After the above tests, the validity and superiority of RBA have been verified. With the increase of environment complexity, the path planning effect of RBA is basically not influenced, and the optimal path can be realized in a relatively short time. Simultaneously, the robustness of RBA has also been proven, and it has good adaptability in complex environments.

## Real-world case

Except for the above simulation experiments, real-world experiments are carried out to verify the real-time performance and effectiveness of our algorithm. The TurtleBot 2 mobile robot equipped with SLAMTEC RPLIDAR A3 is adopted as the experimental platform. In addition, the motion commands of the robot are generated by an IRU-K10 minicomputer with Ubuntu 16.04 and ROS Kinetic installed. The experimental environment map, robot localization and robot path planning are implemented by ROS packages *gmapping*, *amcl* and *move_base*, respectively.

**Fig 10:**
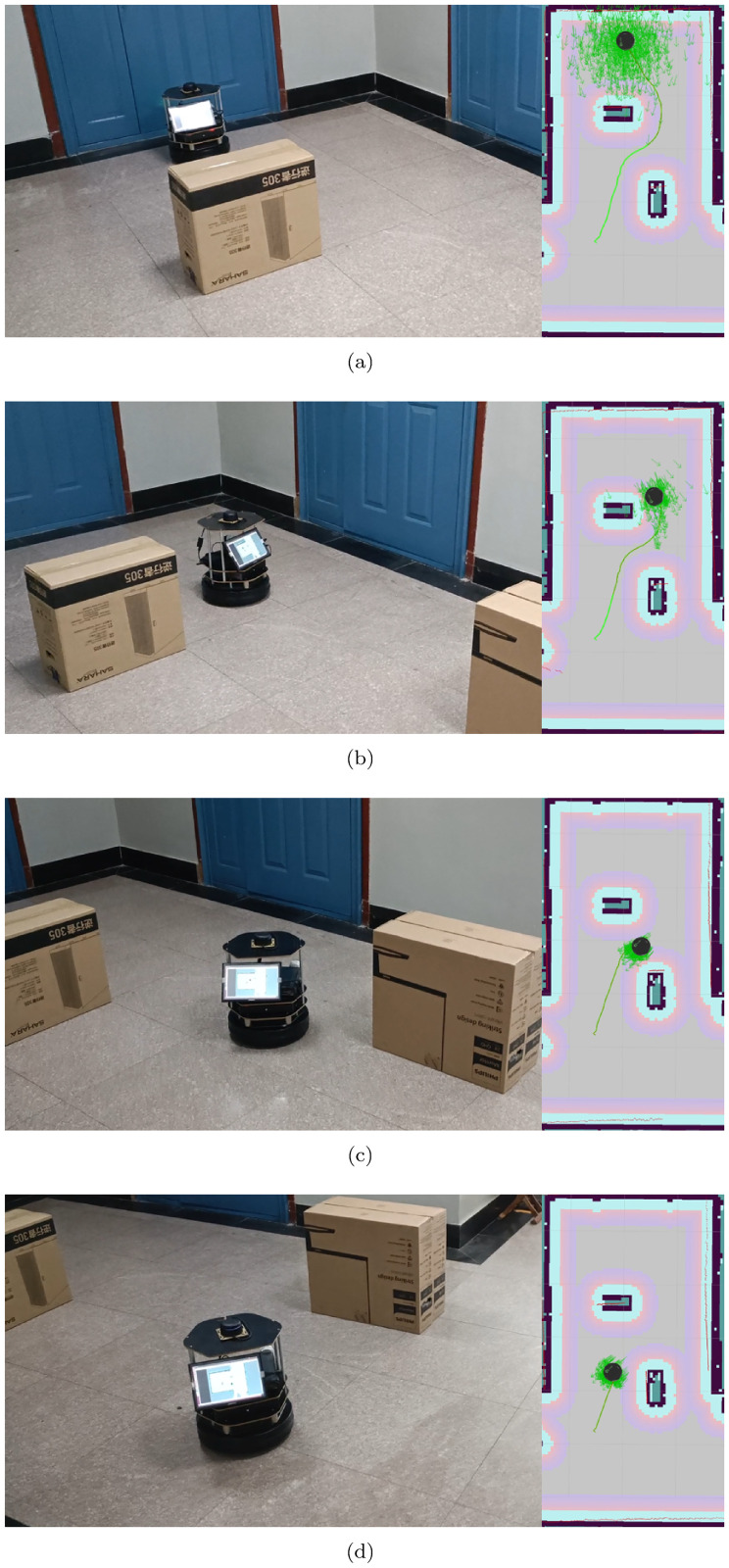
Real-world robot navigation.

The real-world experimental results are depicted in Fig 10, where the black circle indicates the robot, the green line is the global path of the robot planned by the proposed algorithm RBA, and the green arrow cluster is the particle cloud, representing the robot pose estimated by *amcl*. It is evident from Fig 10 that RBA can plan the global optimal path for the robot in real time. Additionally, along this path, the robot can reach the target point safely and efficiently, thus confirming the feasibility and validity of the proposed algorithm.

## Conclusions and future work

In this study, a reformative BA is put forth and effectively addresses the mobile robot path planning problem, mainly relying on the following contributions. First, the Doppler effect is applied to frequency update to ameliorate RBA. When the robot is in motion, the Doppler effect can be adaptively compensated to prevent the robot from prematurely converging. Second, the chaotic map and dynamic disturbance coefficient are adopted in the velocity update and position update respectively to weaken the limitation of local optimum and expand the scope of global exploration. Third, Q-learning is integrated into RBA to make reasonable choices for the loudness attenuation coefficient and the pulse emission enhancement coefficient to optimize the algorithm performance and improve the local exploitation capability. Various simulation results verify the effectiveness and superiority of RBA. Compared with other algorithms, RBA has good comprehensive performance in path planning tasks. Furthermore, as the complexity of the environment increases, RBA exhibits superior robustness, and has the merits of fewer iterations, high success rate and high efficiency. Ultimately, real-world experimental results demonstrate that RBA can accomplish the global optimal path planning in real time, and along the optimal path, the robot can reach the target safely and efficiently.

Nevertheless, our work encompasses the following limitations. On the one hand, only the path planning problem in static scenes is considered, while the influence of dynamic obstacles is ignored. On the other hand, only the single-objective optimization problem is solved, while the multi-objective optimization situation is not comprehensively taken into account. Therefore, based on the above defects, in future work, we will comprehensively consider constraints such as path length, collision risk degree and path smoothness to address the path planning issue of mobile robots in static and dynamic environments.

## Supporting information

S1 Data Code and data. This file provides the relevant code for Test case 1 and Test case 2, as well as the experimental data for Figs 2–4 and 6–8.
(ZIP)
